# Single and multiple dose pharmacokinetics of maritime pine bark extract (Pycnogenol) after oral administration to healthy volunteers

**DOI:** 10.1186/1472-6904-6-4

**Published:** 2006-08-03

**Authors:** Tanja Grimm, Roswitha Skrabala, Zuzana Chovanová, Jana Muchová, Katarína Sumegová, Anna Liptáková, Zdeňka Ďuračková, Petra Högger

**Affiliations:** 1 Institut für Pharmazie und Lebensmittelchemie, Bayerische Julius-Maximilians-Universität, Würzburg, Germany; 2 Department of Medical Chemistry, Biochemistry and Clinical Biochemistry, Comenius University, Faculty of Medicine, Bratislava, Slovakia

## Abstract

**Background:**

Since plant extracts are increasingly used as phytotherapeutics or dietary supplements information on bioavailability, bioefficacy and safety are warranted. We elucidated the plasma kinetics of genuine extract components and metabolites after single and multiple ingestion of the standardized maritime pine bark extract Pycnogenol (USP quality) by human volunteers.

**Methods:**

Eleven volunteers received a single dose of 300 mg pine bark extract, five volunteers ingested 200 mg daily for five days to reach steady state concentrations. Plasma samples were obtained before and at defined time points after intake of the extract. Samples were analyzed by HPLC with ion-pair reagents and simultaneous UV and electrochemical detection.

**Results:**

We quantified total plasma concentrations of catechin, caffeic acid, ferulic acid, taxifolin and the metabolite M1 (δ-(3,4-dihydroxy-phenyl)-γ-valerolactone). Additionally, we describe plasma time courses and steady state appearance of ten so far unknown compounds, U1 to U10. After single ingestion, compounds derived from the extract were rapidly absorbed and the majority of them were detectable over whole experimental period of 14 h. The analysis of steady state plasma samples revealed significant phase II metabolism.

**Conclusion:**

We present the first systematic pharmacokinetic analysis of compounds derived from maritime pine bark extract. Beyond the known constituents and metabolites we uncovered the plasma time courses of ten unknown compounds. In concert with our previous detection of anti-inflammatory bioefficacy of these plasma samples *ex vivo *we suggest that constituents and metabolites of Pycnogenol bear potential for disclosure of novel active principles.

## Background

Since plant extracts are increasingly used as phytotherapeutics or dietary supplements information on bioavailability, bioefficacy and safety are warranted. Safety studies concerning herbal extracts mostly focus on their impact on metabolic enzyme activity and interactions with defined chemical drugs [1,2]. Bioefficacy of plant extracts is progressively investigated in human intervention studies [3], although there is room for improvement regarding methodological quality, sample size, and number of trials [4]. Bioavailability studies are essential completions for the understanding of risk-benefit values and mode of action of herbal remedies. Investigations of kinetics and extent of extract absorption in humans usually focus on a single or some few known lead compounds [5,6]. Only a few studies consider kinetics after single and repeated intake of the respective herbal extract [7,8].

One potential difficulty for the elucidation of both bioavailability and physiological effects of plant extracts is their varying composition that derives from production under different protocols or procedures. Therefore, standardization of the extract is the basis of a reproducible results and effects that relate to batch, preparation, variety and species of the plant [9]. One approach towards quality control of plant extracts is the description of tests, analytical procedures, and acceptance criteria in monographs of internationally accepted pharmacopeias, such as the United States Pharmacopeia (USP). The USP provides the latest FDA-enforceable standards of quality, identity, strength, and purity for drug ingredients, dosage forms, medical devices, and recently also various plant extracts.

An extract with quality specified in the USP 28 is maritime pine bark extract [10]. A standardized bark extract that complies with this monograph is derived from of *Pinus pinaster, Ait*., (Pycnogenol^®^, Horphag Research Ltd., UK). About 65–75 % of the Pycnogenol extract are procyanidins that consist of catechin and epicatechin subunits of varying chain lengths [11]. Other constituents are polyphenolic monomers, phenolic or cinnamic acids and their glycosides.

The procyaninidine-rich maritime pine bark extract Pycnogenol exhibited diverse pharmacological actions in human trials [11]. These effects include, but are not limited to cardiovascular or anti-inflammatory bioefficacy. After oral administration to human patients Pycnogenol exerted effects on circulatory functions such as inhibition of platelet aggregation, a moderate antihypertensive effect and improved microcirculation [11,12]. Anti-inflammatory effects of maritime pine bark extract were observed in asthma patients [13,14].

Though clinical effects of Pycnogenol have been documented little is known about absorption of extract constituents and their metabolism. After oral administration of Pycnogenol ferulic acid and taxifolin were detected in urine after treatment of samples with sulfatase and glucuronidase [15]. Both taxifolin and ferulic acid are genuine components of Pycnogenol. In the same experimental setting two metabolites that were not originally present in the pine bark extract were identified as δ-(3,4-dihydroxy-phenyl)-γ-valerolactone and δ-(3-methoxy-4-hydroxy-phenyl)-γ-valerolactone [15]. Free and conjugated ferulic acid was also detected in urine samples of volunteers after intake of Pycnogenol in another study [16]. The authors suggested ferulic acid as a marker compound for consumption of maritime pine bark extract.

So far, however, no information regarding the time course of constituents' or metabolites' concentration of Pycnogenol in plasma was available. The purpose of the present study was to elucidate the kinetics and rate of absorption of genuine extract components and metabolites after single and multiple ingestion of the pine bark extract by human volunteers. Thereby, we aimed to describe the time course of known and any so far unknown compounds to generate basic information for understanding bioavailability and bioefficacy of Pycnogenol.

## Methods

### Volunteers

Healthy female and male volunteers aged 18 to 30 years participated in this study. Both study protocols were approved by the ethical committee of the Comenius University's Faculty of Medicine, Bratislava, Slovak Republic, and all participants gave written informed consent.

### Protocol of single intake of Pycnogenol

Eleven volunteers (five female and six male) participated in this study. After a 24 hour diet free of flavonoids (no vegetables, fruits and fruit juices or marmalades, tea, coffee, cocoa, wine and beer) a venous blood catheter was inserted into an antecubital vein and blood samples were drawn to obtain basal values (t = 0 h at 8:00 a.m.). Subsequently, the volunteers received a single dose of six 50 mg tablets (preparation of study medication by DKSH, Market intelligence, Tokyo, Japan) containing 300 mg standardized maritime pine bark extract (Pycnogenol^®^, Horphag Research Ltd., UK) with 200 mL tap water. At 8:15 a.m. the volunteers had a standardized breakfast (two white rolls (bread) and 0.3–0.5 L of milk (1.5 % fat)), at 12:15 lunch (1/4 of only salted and baked chicken with white bread) and at 6:15 p.m. dinner (100 g ham, 50 g cheese (eidam) and bread with butter or margarine). After each blood sampling mineral water was served. Blood samples were obtained at t = 0.5 h, 1 h, 2 h, 4 h, 6 h, 8 h, 10 h, 12 h and 14 h. Samples were centrifuged and plasma was aliquoted, shock frozen and stored at -80°C until further analysis.

### Protocol of repeated intake of Pycnogenol

Five volunteers (four female and one male) participated in this study. After a 24 hour diet free of flavonoids blood samples were drawn to obtain basal values. Subsequently, the volunteers took four 50 mg tablets containing 200 mg Pycnogenol every morning for five days to reach steady state conditions of constituents and/or metabolites of Pycnogenol. It was assumed that steady state plasma concentrations were reached after 5 days.

Four hours after the last intake of Pycnogenol on day five a second blood sample was obtained from each volunteer. Again, a 24 hour period of a diet free of flavonoids preceded this blood sampling. Blood samples were centrifuged and plasma was aliquoted, shock frozen and stored at -80°C until further analysis.

### Chemicals and reagents

A spray-dried extract from maritime pine bark (Pycnogenol^®^) was generously provided by Horphag Research Ltd. (Geneva, Switzerland). The metabolites M1 (δ-(3,4-Dihydroxy-phenyl)-γ-valerolactone) and M2 (δ-(3-Methoxy-4-hydroxy-phenyl)-γ-valerolactone) were synthesized by Groβe Düweler[15] The monomeric compounds (+)-catechin, (-)-epicatechin, ferulic acid, gallic acid, 4-hydroxybenzoic acid, caffeic acid, protocatechuic acid, sinapic acid und (±)-taxifolin were purchased from Sigma-Aldrich (St. Louis, MO, USA). All other chemicals were obtained from Sigma-Aldrich or Merck (Darmstadt, Germany), if not stated otherwise. All chemicals used were of highest purity available.

Krebs-Ringer-HEPES buffer (pH 7.4) consisted of 118 mM NaCl, 4.84 mM KCl, 1.2 mM KH_2_PO_4_, 2.43 mM MgSO_4_, 2.44 mM CaCl_2 _× 2 H_2_O and 10 mM HEPES.

### Preparation of plasma samples

Plasma samples of 2.0 mL were prepared for analysis. For determination of total plasma concentration, to each sample 20 U β-glucuronidase (type H-3 from Helix pomatia; EC 3.2.1.31) and 20 U sulfatase (type H-1 from Helix pomatia; EC 3.1.6.1) were added in 50 mM sodium acetate buffer (pH 5.0) and incubated for 2 h at 37°C under gentle shaking. Samples for determination of free plasma concentrations were prepared without prior incubation with enzymes. Each 20 μL of p-hydroxybenzoic acid methylester (100 μg/mL; for HPLC UV detection) and hydrochinone (10 μg/mL; for HPLC electrochemical detection) were added as internal standards and samples were acidified with 100 μL 1 M-HCl. Samples were extracted twice with each 3 mL acetic acid methylester for 20 min, using a roller mixer, followed by centrifugation (20°C) 5 min. The organic phases of both extractions were separated, combined and evaporated to dryness under a gentle stream of nitrogen at 25°C. The resulting residue was reconstituted in 100 μL methanol and subjected to HPLC analysis.

Calibration curves of all known compounds of Pycnogenol were prepared by addition of the respective substances to 2.0 mL of Krebs-Ringer-HEPES buffer (pH 7.4). This was necessary because sufficient volumes of pooled blank plasma of blood donors who kept a 24 hour diet free of flavonoids was not available (total volume needed was about 800 mL for all experiments). Thus, buffer instead of plasma was used and treated analogously as plasma samples from volunteers. Since initial control experiments revealed that the incubation with β-glucuronidase and sulfatase did not produce interfering peaks in the HPLC chromatograms no enzymes were added to calibration curve samples.

### Analysis of plasma samples by HPLC – UV/electrochemical dual detection

The HPLC system was a Waters HPLC (Milford, MA, USA) consisted of a 1525 binary pump, a 717plus autosampler, a 2487 dual wavelength absorbance detector set at the detection wavelength of 280 nm and an electrochemical detector CLC 100 (Chromsystems, Munich, Germany) set at an oxidation voltage of 0.5 V. The second detector was connected to the control system by a satellite interface (Waters). Data collection and integration were accomplished using Breeze™ software version 3.30. Analysis was performed on a Zorbax SB C_8 _column (150 × 4.6 mm I.D., 5 μm particle size, Agilent Technologies, Palo Alto, CA, USA).

Typically, 20 μL of sample were injected and separated at a flow rate of 1 mL/min. Isocratic elution was performed using water (containing 0.6 mM 1-octanesulfonic acid sodium salt, 0.27 mM ethylenediaminetetraacetic acid disodium salt, 0.04 M triethylamine; pH 2.95 adjusted with phosphoric acid) and acetonitrile (ACN, HPLC gradient quality, Fisher Scientific, Schwerte, Germany). Method A (for UV detection of ferulic acid, M2 (δ-(3-Methoxy-4-hydroxy-phenyl)-γ-valerolactone), U1, U2, U3, U4, U7, U8, and U9) used water/ACN at 85:15 (v/v). Method B (for electrochemical detection of catechin, caffeic acid, taxifolin, M1 (δ-(3,4-dihydroxy-phenyl)-γ-valerolactone), U1, U2, U4, U5, U6, U8, U9 and U10) used water/ACN at 88:12 (v/v).

The analytical method was validated according to ICH guidelines. The method fulfilled the quality criteria for selectivity, linearity, precision and accuracy. The calibration curves' working range was 0.5–20 ng/mL for caffeic acid, 1–50 ng/mL for M1, 10–100 ng/mL for catechin, taxifolin and ferulic acid, 50–200 ng/mL for M2. The recovery rates after extraction were 83.1 % (M2) to 98.0 % (taxifolin). The lower limits of quantitation were 0.5 ng/mL for caffeic acid, 1 ng/mL for M1, 10 ng/mL for catechin, taxifolin and ferulic acid, 50 ng/mL for M2.

### Quantitation of pine bark extract constituents in tablets by HPLC – UV detection

Pycnogenol tablets prepared for this study (see above) were pounded and suspended in methanol to yield concentrations of 1 mg/mL, vortexted for 2 minutes, centrifuged and the supernatant collected. The procedure was repeated 4 times. The amount of taxifolin, ferulic acid, caffeic acid and catechin in the Pycnogenol tablets administered to the volunteers was calculated on the basis of calibration curves. They were constructed with five concentrations ranging from 5–20 μg/mL for taxifolin, from 1–10 μg/mL for ferulic acid, and from 0.1–8 mg/mL for caffeic acid. Analysis was performed on an Atlantis C-18d column (150 × 3.9 mm i.d, 3 μm particle size; Waters). The mobile phase consisted of water containing 1% acetic acid (A) and acetonitrile (B). The gradient elution started at 98 % eluent A increasing linearly to 84 % within 60 minutes and a flow rate of 1 mL/min. The UV detection wavelength was set to 280 nm.

## Results

### Detection of Pycnogenol constituents and metabolites in plasma

After oral ingestion of either a single dose of 300 mg or multiple doses of 200 mg plasma concentrations of constituents or metabolites of Pycnogenol were detectable in all volunteers without exception. Unless otherwise indicated, plasma levels repesent the sum of free and conjugated compounds. After 300 mg single dose intake a mean of 11 ± 2 (range 7–14) different constituents or metabolites were found in the plasma samples of each volunteer. After 200 mg multiple dose intake a mean of 7 ± 2 (range 4–10) different constituents or metabolites were detected in the plasma sample of each volunteer. A total of 15 different compounds were detectable after single dose intake. Of those substances detected after single dose intake, 12 compounds were also found in plasma samples after multiple dose ingestion.

**Figure 1:**
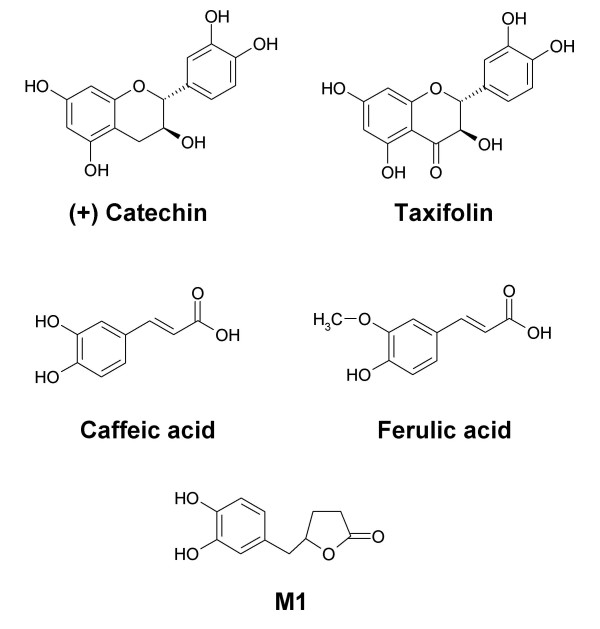
Structural formulas of the known compounds that were detected in plasma samples of volunteers after intake of Pycnogenol.

Of the 15 compounds that were present in plasma samples 5 were known constituents or metabolites of Pycnogenol (catechin, caffeic acid, ferulic acid, taxifolin and M1 (δ-(3,4-dihydroxy-phenyl)-γ-valerolactone), Figure 1). The other 10 compounds were unknown, they were not identical with any previously described component of maritime pine bark extract, e.g. coumaric acid, (-)-epicatechin, gallic acid, 4-hydroxybenzoic acid, protocatechuic acid or M2 (δ-(3-Methoxy-4-hydroxy-phenyl)-γ-valerolactone). The 10 unknown compounds were labelled as U1 to U10 according to their retention time at the reversed-phase HPLC column. The compound U1 was eluted after 4.5 min, U2 after 5.4 min, U3 after 13.4 min, U4 after 14.3 min, U5 after 15.5 min, U6 after 16.2 min, U7 after 17.2 min, U8 after 20.8 min, U9 after 22.2 min and U10 after 35.8 min. For all unknown substances the peak area ratios of the respective compound to the internal standard were calculated which allowed graphical representation of the time course in plasma.

### Pycnogenol constituents and metabolites in plasma after a single dose of 300 mg

#### Compounds with t_max _up to 5 hours

**Figure 2:**
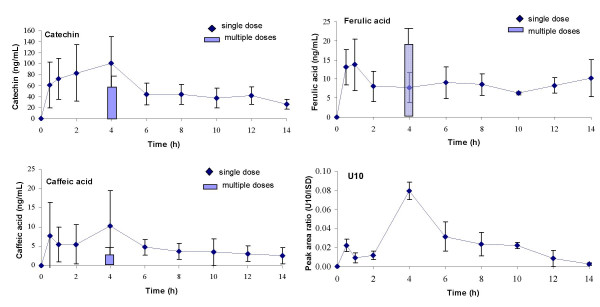
Time course of total (free and conjugated) plasma concentrations of catechin, caffeic acid, ferulic acid and U10. These compounds revealed an early t_max _(time of maximal plasma concentration) up to five hours after intake of the pine bark extract. Symbols represent time course of mean and standard deviation of concentrations after a single dose of 300 mg Pycnogenol. The columns represents mean and standard deviation of concentrations after repeated doses of 200 mg Pycnogenol daily after five days (assumed steady state). Steady state concentrations of U10 were detectable in only two volunteers and are not shown in this diagram.

Four compounds found in the plasma samples after single intake of 300 mg Pycnogenol displayed early maximum concentrations up to five hours and were measurable over the whole time period of 14 h (Figure 2). Catechin was detectable rapidly with mean concentrations of about 60 ng/mL after 0.5 h already. Thereafter, the time course revealed gradually increasing concentrations to about 100 ng/mL after 4 h and subsequent decrease of plasma levels. However, catechin was detectable over the whole period of time. The plasma concentrations were almost constant from 6 to 14 h. A similar time course, but significantly lower of plasma concentrations were seen for caffeic acid. Maximum plasma concentrations of ferulic acid were seen already after 0.5 to 1 h. Concentrations decreased thereafter and remained almost constant before revealing another increase towards the end of experimental period. The unknown compound U10 was present in the plasma samples after 0.5 h as well, but maximum concentrations were reached not before 4 h. Thereafter, the plasma levels steadily decreased and U10 appeared to be almost eliminated after 14 h.

#### Compounds with t_max _between 5 and 10 hours

**Figure 3:**
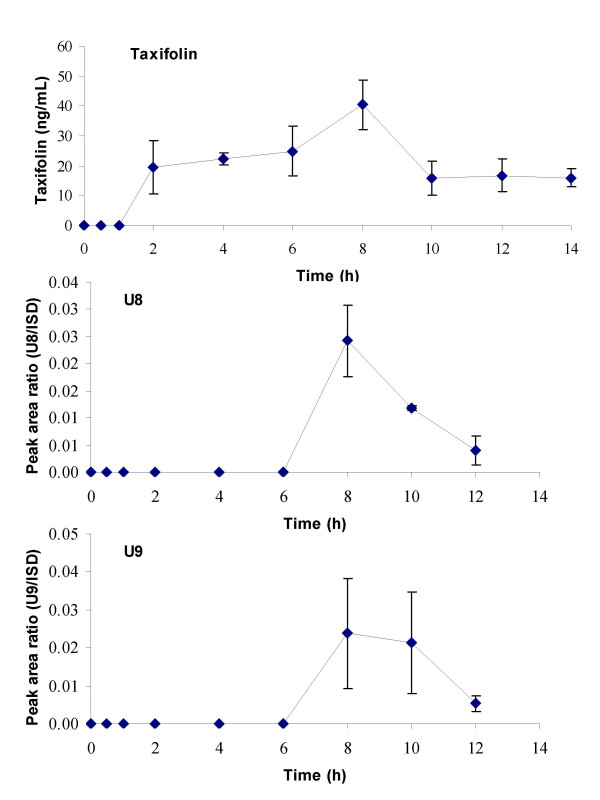
Time course of total (free and conjugated) plasma concentrations of taxifolin, U8 and U9. These compounds had a t_max _between five and ten hours. Symbols represent time course of mean and standard deviation of concentrations after a single dose of 300 mg Pycnogenol. No steady state concentrations after repeated doses of 200 mg Pycnogenol daily were detectable after five days.

Three compounds displayed maximum plasma levels between 5 and 10 h after intake of Pycnogenol tablets (Figure 3). Taxifolin was not detectable before 2 h, maximum concentrations were recorded after 8 h. Thereafter, the taxifolin levels remained almost constant until the end of the experimental period. Both unknown compounds U8 and U9 appeared rather late after 6 h in plasma and both were rapidly eliminated with no measurable concentrations after 14 h.

#### Compounds with t_max _around 10 hours

**Figure 4:**
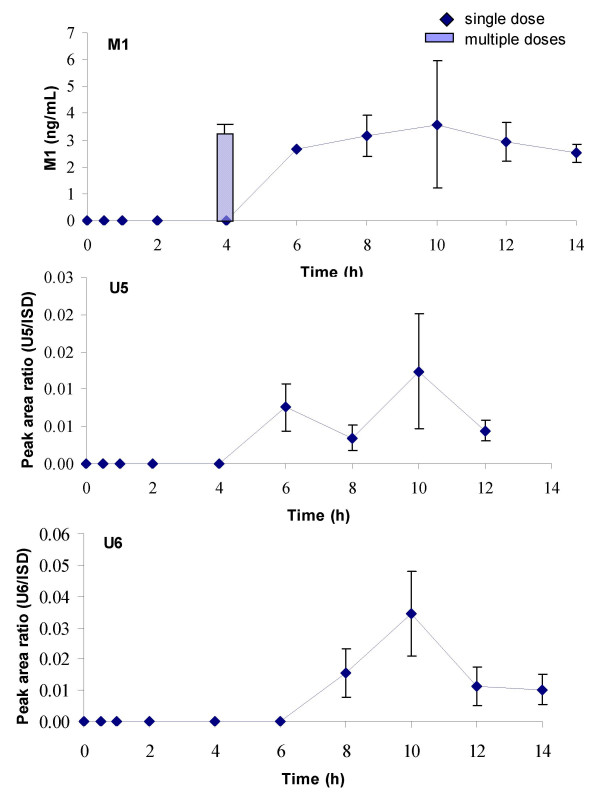
Time course of total (free and conjugated) plasma concentrations of M1, U5 and U6. These compounds had a t_max_around ten hours. Symbols represent time course of mean and standard deviation of concentrations after a single dose of 300 mg Pycnogenol. The column represents mean and standard deviation of M1 concentrations after repeated doses of 200 mg Pycnogenol daily after five days. Steady state concentrations of U5 and U6 were detectable in one, respectively two volunteers and are not shown in this diagram.

Three compounds showed late maximum plasma levels 10 h after intake of Pycnogenol tablets (Figure 4). One was the previously identified metabolite M1 (δ-(3,4-dihydroxy-phenyl)-γ-valerolactone) that has been found in urine samples before [15]. Consistent with the notion that it is formed *in vivo *from catechin by bacterial metabolism this substance appeared late in plasma. It was detectable after 6 h, revealed a peak concentration around 10 h (mean concentration 3.59 ng/mL) and was still present 14 h after Pycnogenol ingestion. Two further compounds of unknown identity displayed a similar time course with a t_max _around 10 h, but the plasma concentrations of U5 and U6 decreased towards the end of the experimental period.

#### Compounds with interindividually highly variable t_max_

**Figure 5:**
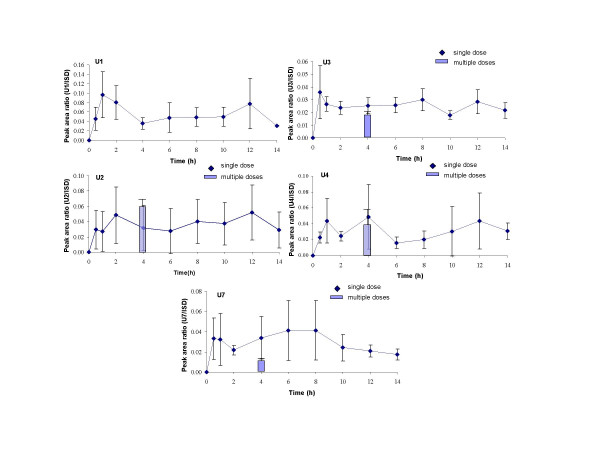
Time course of total (free and conjugated) plasma concentrations of U1, U2, U3, U4 and U7. These compounds revealed high interindividual variability of the t_max_. Symbols represent time course of mean and standard deviation of concentrations after a single dose of 300 mg Pycnogenol. The columns represent mean and standard deviation of concentrations after repeated doses of 200 mg Pycnogenol daily after five days. Steady state concentrations of U1 were detectable in only two volunteers and are not shown in this diagram.

Five unknown compounds revealed time courses after intake of Pycnogenol with pronounced interindividual variability in time of maximum concentrations (Figure 5). When mean concentrations were calculated, the mean time course thus appeared to display either no pronounced or multiple t_max_, e.g. apparently three t_max _for U4. However, when the time course of the plasma concentrations of individual volunteers was inspected, it revealed always two t_max_, one early t_max _between 0.5 to 2 h and one late t_max _between 8 and 12 h. All five substances, U1 to U4 and U7 were detectable over the whole experimental period. All were readily present in the plasma samples, especially U3 and U7 revealed high concentrations 0.5 h after ingestion of Pycnogenol already.

### Pycnogenol constituents and metabolites in plasma after multiple doses of 200 mg

Most constituents or metabolites that were detected in the volunteers' plasma samples after intake of a 300 mg single dose were also found after repeated intake of 200 mg Pycnogenol (columns in Figures 2, 3, 4, 5). Plasma samples were obtained 4 h after the last dose of Pycnogenol. It was assumed that steady state conditions were reached after five days ingestion of the pine bark extract. Steady state concentrations of all compounds except for taxifolin, U8 and U9 were detectable at least in one volunteer. However, only when steady state concentrations of a constituent or metabolite in the plasma of at least three volunteers were measurable, these are shown as columns in the graphical plots. Thus, no data are shown for U1 (two plasma samples with measurable concentrations), U5 (one sample), U6 (two samples), U10 (two samples). Plasma concentrations of taxifolin, U8 and U9 were below the limit of quantitation for all volunteers after the multiple doses of 200 mg. For most compounds the mean steady state plasma concentration was well within the range of individual variability in concentrations measured after single ingestion of the pine bark extract. One exception is the metabolite M1 which revealed mean steady state plasma concentrations (3.01 ng/mL) which were similar to concentrations measured after single intake of Pycnogenol.

### Individual degree of conjugation with sulfate and glucuronic acid

The plasma samples obtained after repeated intake of 200 mg Pycnogenol daily were additionally analyzed for the degree of conjugation with sulfate and glucuronic acid. This type of phase II metabolism has been described for Pycnogenol constitutents and metabolites [15,16]. Generally, the chomatograms of plasma samples that were not pre-incubated with sulfatase and glucuronidase revealed less and smaller compound peaks. The degree of conjugation displayed significant interindividual variability. Data of compounds that revealed detectable total plasma concentrations and a degree of conjugation less than 100 % are listed in Table 1. For catechin, the range was 0 % conjugated (volunteer 4) to ≈ 100 % conjugated (volunteer 3). The other volunteers revealed 43.3 %, 66.8 % and 72.3 % conjugation of catechin (mean percentage of conjugation 56.5 %). Three volunteers had detectable plasma concentrations of free caffeic acid. The individual degree of conjugation was 59.6 %, 61.5 % and 87.6 % (mean 69.4 %). The unknown compound U2 was highly conjugated; two volunteers' samples were ≈ 100 % conjugated, the other 32.4 %, 68.5 % and 84.7 % (mean 77.1 %). Only three volunteers had detectable plasma concentrations of U7. The individual degree of conjugation was 17.3 %, 46.3 % and 71.4 % for U7 (mean 45 %). Total plasma concentrations of M1 (n = 3), ferulic acid (n = 3), U1 (n = 2), U3 (n = 3), U4 (n = 4), U5 (n = 1), U6 (n = 2) and U10 (n = 2) were detectable in some of the volunteers. Since no free concentrations were found we conclude that these compounds were about 100 % conjugated. Plasma concentrations of taxifolin, U8 and U9 were below the lower limit of quantitation in all volunteers.

**Table 1:** Detectable plasma concentrations of compounds that revealed a mean degree of phase II conjugation of less than 100 %: catechin (n = 5), caffeic acid (n = 3), U2 (n = 5) and U7 (n = 3) in samples of volunteers after repeated intake of 200 mg Pycnogenol once daily for five days.

	Total plasma concentration	Free plasma concentration	% conjugated
Catechin [ng/ml]	48.6 ± 16.7	21.0 ± 13.1	56.5 ± 27.9
Caffeic acid [ng/ml]	2.42 ± 1.39	0.56 ± 0.02	69.4 ± 11.8
U2 [peak ratio]	0.0537 ± 0.0132	0.0105 ± 0.0084	77.1 ± 21.3
U7 [peak ratio]	0.0101 ± 0.0007	0.0057 ± 0.0020	45.0 ± 18.5

Plasma concentrations were determined with and without prior incubation of sulfatase and glucuronidase (total and free plasma concentration, respectively) and individual percentage of phase II conjugation was calculated. Mean concentrations and mean deviations of the mean are listed.

### Analysis of Pycnogenol in tablets used in the study

The quality of the Pycnogenol tablets used in this study was analyzed according to the USP 28 monograph [10]. The tablets were in conformity with the acceptance criteria defined in the monograph. In order to calculate pharmacokinetic parameters it was necessary to quantify the content of known single nonconjugated extract components. Therefore, the tablets were subjected to HPLC analysis and content of single compounds was calculated based on calibration curves with the pure compound. The calculated content was 1.75 μg free caffeic acid per mg extract in the study medication, 3.25 μg free ferulic acid per mg extract and 14.35 μg free taxifolin per mg Pycnogenol.

### Pharmacokinetic parameters of compounds detected after single dose intake

From individual time courses after administration of 300 mg Pycnogenol as a single dose pharmacokinetic parameters were calculated. Mean and standard deviations of individual results were summarized (Table 2). For caffeic acid (calculated dose administered: 0.525 mg), taxifolin (calculated dose: 4.31 mg) and ferulic acid (calculated dose: 0.975 mg) the AUC, c_max_, t_max _and terminal t½ were determined. These calculations were based on the determined free concentration of the respective compound in the extract and based on the assumption that no additional caffeic or ferulic acid was metabolically generated. For both catechin and metabolite M1 only c_max _and t_max _were calculated. Since unknown amounts of additional catechin besides genuinely contained monomeric catechin might be generated by metabolic breakdown of higher procyanidin oligomers [17] no additional parameters could be determined. Likewise, because M1 is generated from catechin units [15,17] the dose administered was unknown.

**Table 2:** Calculated pharmacokinetic parameters of components or metabolites after intake of a single dose of 300 mg Pycnogenol.

Compound	n	AUC_ [0-t] _[ng/mL × h]	AUC_ [t-8] _[ng/mL × h]	c_max _[ng/mL]	t_max _[h]	term t½ [h]
Caffeic acid	9	75.66 ± 33.53	82.78 ± 36.47	16.67 ± 13.29	3.7 ± 2.4	4.42 ± 2.47
Catechin	9			107.22 ± 55.49	3.2 ± 1.7	
Taxifolin	6	231.11 ± 85.98	399.14 ± 98.95	33.34 ± 12.54	8.2 ± 2.5	8.89 ± 2.81
Ferulic acid	7	99.05 ± 28.09	141.19 ± 73.90	14.78 ± 5.89	1.2 ± 1.1	6.87 ± 3.83
M1	8			4.11 ± 2.08	10.0 ± 1.9	

Abbreviations: n – number of volunteers with detectable compound concentrations; AUC – area under the curve, "t" symbolizes the time of last plasma sampling (14 h); c_max _– maximal plasma concentration, t_max _– time of maximal plasma concentration; term t½ – terminal half-life of compound. Values represent means and standard deviations. These calculations were based on the determined free concentration of the respective compound in the extract and based on the assumption that no additional caffeic or ferulic acid was metabolically generated.

## Discussion

In the present investigation we present the results of an extensive analysis of plasma concentrations of constituents and metabolites of standardized maritime pine bark extract (Pycnogenol) after single and multiple administrations to healthy human volunteers. After ingestion, compounds derived from the extract were absorbed by all volunteers. We quantified the plasma concentrations of catechin, caffeic acid, ferulic acid, taxifolin and the metabolite M1 (δ-(3,4-dihydroxy-phenyl)-γ-valerolactone). Additionally, we describe plasma time courses and steady state appearance of ten so far unknown compounds, U1 to U10.

The oral bioavailability of molecules with drug-like properties depends on their physicochemical properties which have been described as the "rule of five" [18]. Absorption is most probable if a compound has less than 5 hydrogen bond donors, less than 10 hydrogen acceptors, a relative molecular mass below 500 and a logP smaller than 5. Exceptions from this rule are known for many orally active drugs which often share structural similarity with substrates of specific transporters which enable enhanced absorption. All monomeric polyphenols detected in plasma samples in the present study comply with the "rule of five" and thus their bioavailability might not appear surprising. However, since these genuine compounds of the maritime pine bark extract represent only a small fraction of the whole extract their presence in plasma samples substantiates that the excess of higher procyanidin oligomers does not interfere with absorption of smaller molecules. Plasma concentrations of caffeic acid, ferulic acid and taxifolin were well detectable although the calculated intake of the respective compounds was only between 0.5 and 4 mg after a 300 mg Pycnogenol dose.

Catechin levels were readily detectable in plasma samples of the volunteers after single and multiple intake of Pycnogenol as well. It was not possible to determine the administered "dose" of catechin. Catechin is as well genuinely present in the extract as polyphenolic monomer and might be additionally generated by metabolic breakdown of higher procyanidin oligomers [17]. In the present study the catechin plasma concentration peaked with about 100 ng/mL (≈ 370 nmol/L) within the first 3 h. Maximal plasma concentrations of catechin were observed after 1.5 h in human plasma after ingestion of red wine or reconstituted red wine [19,20] and 0.5 h or 2–3 h after intake of pure catechin [21,22]. Thus, the t_max _of catechin observed in the present investigation is within the range described in other studies. In contrast to the time course of plasma levels after ingestion of the pure compound, we observed catechin over the whole experimental period and the levels remained almost constant between 6 h and 14 h. This is consistent with metabolic generation of catechin [17] monomers from oligomeric procyanidins. By 4 h after ingestion of pure catechin the plasma concentrations were not significantly different from baseline levels [22]. After a high single dose the unchanged catechin was detected in plasma between 30 min and 12 h after administration, metabolites for a shorter period of time [21]. After a single dose of pure catechin the free polyphenol concentration was only 1.1 to 6.5 % of the total concentration. We observed higher free catechin concentrations of about 56 % after multiple intake of maritime pine bark extract, but the interindividual variability was high.

Caffeic acid was present in the volunteers' plasma samples already 0.5 h after ingestion of Pycnogenol. This fast systemic availability is consistent with other reports [6,23-25]. While we determined a t_max _of caffeic acid between 3–4 h others report t_max _values between 0.5–1 h after intake of red wine, coffee or artichoke leaf extract [6,24,25] or a t_max _2 h after ingestion of red wine [23]. Though the t_max _we observed appears to be slightly later than in other investigations the maximal plasma concentrations we measured were comparatively high. After a calculated intake of 525 μg caffeic acid a c_max _about 17 ng/mL (≈ 90 nmol/L) was found, which is comparable to a c_max _of 84 nmol/L after intake of 55 μg caffeic acid per kg body weight [23]. After ingestion of 107 mg caffeic acid from artichoke leaf extract a lower c_max _of 6.5 ng/mL was reported [6]. The later t_max _and higher c_max _found in the present study might indicate additional sources of caffeic acid from so far not identified metabolic pathways or due to the presence of additional caffeic acid as conjugate in the pine bark extract. A 5.4 fold increase of caffeic acid concentration in beer after hydrolysis was recently described [26]. The elimination half-live of 4.42 ± 2.47 h we calculated for caffeic acid is consistent with the t½ of 3.08 ± 1.53 h reported previously [6]. The degree of conjugation of caffeic acid we found after multiple intake of the maritime pine bark extract (mean 69 %) was within the range reported after single intake of coffee (around 70–80 %) [25], but lower than after or after beer ingestion (around 90 %) [26].

Ferulic acid has been previously described as a urine excretion marker of consumption of maritime pine bark extract [16] and thus it was expected to be present in plasma. We observed an early t_max _of 1 h for ferulic acid which is consistent with other reports of t_max _values of 1–3 h [27] or 0.77 h [6]. The c_max _of about 15 ng/mL (≈ 75 nmol/L) we observed after intake of the single dose Pycnogenol containing 975 μg free ferulic acid again appears to be high. After ingestion of 250 mg ferulic acid from breakfast cereals plasma concentrations of 150–210 nmol/L were described [27]. However, additional ferulic acid might be present in the pine bark extract in conjugated form and it is known to be generated *in vivo *by methylation of caffeic acid [6,16]. The fact that the plasma concentrations of ferulic acid are almost constant between 2 h and 14 h favour the notion of additionally generated ferulic acid, e.g. from caffeic acid. The remarkably high steady state concentrations of ferulic acid (about 20 ng/mL) after repeated intake of Pycnogenol along with the rather low steady state concentrations of caffeic acid furthermore support the concept of metabolic generation of ferulic acid. The elimination half-live of ferulic acid we calculated (6.87 ± 3.83 h) is completely consistent with the t½ of 6.35 ± 2.95 h reported by Wittemer et al. [6]. In our study we did not detect any free, only conjugated ferulic acid after repeated intake of maritime pine bark extract whereas around 25 % free ferulic acid was found after single ingestion of beer [26].

Taxifolin was detected not before 2 h after ingestion of the pine bark extract and maximal plasma concentrations were recorded after 8 h. Furthermore, no steady state concentrations of conjugated or free taxifolin were detectable after multiple intake of Pycnogenol. This plasma concentration profile of taxifolin was quite unexpected since free taxifolin is present in the extract at higher concentrations than other polyphenol monomers. To our knowledge, no plasma concentrations of taxifolin have been reported for humans before. We supposed that the compound's late appearance in plasma and the lack of steady state levels is due to metabolic degradation processes. The anaerobic bacterium *Clostridium orbiscindens *was found in human feces recently [28]. This bacterium has the ability to degrade taxifolin to 3,4-dihydroxyphenylacetic acid and phloroglucin. However, we failed to detect either of these metabolites in the volunteers' plasma samples.

The catechin metabolites M1 (δ-(3,4-dihydroxy-phenyl)-γ-valerolactone) and M2 (δ-(3-Methoxy-4-hydroxy-phenyl)-γ-valerolactone) have been previously found in urine samples after oral intake of maritime pine bark extract [15] and thus we expected their presence in plasma samples as well. However, we only detected the metabolite M1 after intake of a single dose and after repeated ingestion of Pycnogenol. Since c_max _concentrations of M1 were only around 4 ng/mL (≈ 20 nmol/L) after single and repeated dosing we assume that our analytical method was not sensitive enough to detect M2 (lower limit of quantitation: 50 ng/mL). Consistent with the information that M1 is generated from catechin units [15,17] we observed at late t_max _10 h after ingestion of the extract. Peak plasma levels of M1 were observed 5–12 h after ingestion of green tea [29] and earliest appearance of M1 has been recorded after 3 h after green tea ingestion [30].

Along with the above discussed five known constituents or metabolites of Pycnogenol ten so far unknown compounds were found in the volunteers' plasma samples. These substances are not identical to any other known free polyphenol monomer in the maritime pine bark extract. They may, however, be conjugates of monomers or metabolites of higher procyanidin oligomers. It is also conceivable that a procyanidin dimer is among these unknown compounds. The procyanidin B1 has been detected in human plasma samples [31] and this procyanidin is a constituent of Pycnogenol as well. The unknown compound U10 displays a t_max _4 h after ingestion of the extract. Based on this time course it is very likely that U10 is a constituent rather than a metabolite of Pycnogenol. In contrast, it can be assumed that the compounds U8 and U9 which are detectable only between 8 h and 10 h are metabolites which are either rapidly eliminated or further metabolized. Consequently, these are the only compounds besides taxifolin which are not present in the steady states plasma samples of a single volunteer. The compounds U5 and U6 display typical plasma profiles of metabolites as well since they cannot be discovered before 6 h or 8 h, respectively, after ingestion of Pycnogenol. The unknown compounds U1, U2, U3, U4 and U7 were all readily detectable in plasma after 0.5 h and all revealed an essentially biphasic plasma concentration profile. Although the individual t_max _values vary considerably all substances have one early and one late t_max_. Multiple t_max _values are consistent with either enterohepatic circulation of a compound or with successive metabolic generation of additional substance, e.g. by breakdown of higher procyanidin oligomers.

Regarding the plasma time course of constituents or metabolites of Pycnogenol two observations are remarkable. One is that apparently many constituents are bioavailable after oral ingestion and obviously pronounced metabolism occurs. The other fact is that the concentrations of known compounds both after single and multiple dosing are within the ng/mL range translating into nanomolar concentrations. This appears to be very low and reminds of a question brought up by Goldberg et al. [22] whether biologically effective polyphenol concentrations are attainable in humans. We previously elucidated the effects of two Pycnogenol metabolites, M1 and M2, towards inhibition of activity and release of matrix metalloproteinases (MMP) *in vitro *and in cell culture assays [32]. We found that both metabolites were effective in inhibition of MMP-9 release with IC_50 _values of 0.5 μM. In the present study we learned that these concentrations were not detected in the volunteers' plasma samples. Various conclusions are possible; one is that the *in vitro *experiments are irrelevant because concentrations required for e.g. anti-inflammatory effects are not attained *in vivo*. Opposing this view is the fact that Pycnogenol has demonstrated *in vivo *efficacy in various clinical studies [3,11]. Another conclusion might be that various substances act synergistically and thus efficacy is seen at lower levels of the individual compound. However, so far there are no proofs for this. A last conclusion we would like to suggest is that we possibly have been looking for the wrong compounds. While most *in vitro *studies focused on free polyphenolic monomers it has been reported that the conjugates might exert effects as well [33,34]. Our present study furthermore indicates that multiple unknown constituents and/or metabolites are present in plasma samples after ingestion of maritime pine bark extract. These might well be responsible for the pharmacodynamic effects of Pycnogenol. Our recent results support the suggestion that active principles with significant bioefficacy are present in plasma of human volunteers after ingestion of the extract. Aliquots of plasma samples from the volunteers of the present study were previously assayed for their effects towards cyclooxygenase (COX) activity [35]. The plasma samples after multiple intake of Pycnogenol revealed moderate, but no significant inhibition of COX-1 and COX-2. In contrast, plasma samples obtained 0.5 h after ingestion of the extract significantly inhibited both COX enzymes. We also employed the steady state plasma samples in cell culture assays. Although these plasma samples were diluted 1:1 with cell culture medium they still moderately inhibited NF-κB activation and significantly inhibited release of MMP-9 from activated human monocytes [36]. Consequently, we now have to identify the unknown compounds detected after Pycnogenol intake and elucidate their anti-inflammatory activities.

## Conclusion

We present the first systematic pharmacokinetic analysis of constituents and metabolites of the standardized maritime pine bark extract (USP quality) after single and repeated intake by human volunteers. Components of the extract were bioavailable and detectable in the plasma of all subjects. Pharmacokinetic parameter calculated for the so far identified compounds were comparable with results from other studies. In addition, for the first time we describe steady state concentrations of catechin, caffeic acid, ferulic acid and M1 (δ-(3,4-dihydroxy-phenyl)-γ-valerolactone) and present the first taxifolin plasma concentrations in humans. The detection of ten so far unknown bioavailable constituents and metabolites of Pycnogenol reveal potential for uncovering active compounds with anti-inflammatory bioefficacy.

## Competing interests

This work was supported by a research grant of Horphag Research. They had no role in the collection, analysis and interpretation of data or in the writing of the manuscript.

## Authors' contributions

T.G. carried out all experiments with the plasma samples and the data analysis.

R.S. developed and applied the HPLC method for quantitation of the extract components.

Z.C. recruited the volunteers and organized the study, prepared the technical documentation for blood sampling.

J.M. and K.S. took care of the volunteers and performed blood sampling and processed samples according to the protocol.

A.L. prepared the project and processed blood samples.

Z.D. contributed to planning of the design and execution of the project and wrote the ethic's committee application.

P.H. conceived of and designed the study and wrote the manuscript.

All authors read and approved the final manuscript.

## Pre-publication history

The pre-publication history for this paper can be accessed here:


